# Effectiveness of application of a manual for improvement of alarms management by nurses in Intensive Care Units

**DOI:** 10.17533/udea.iee.v39n2e11

**Published:** 2021-06-15

**Authors:** Amirhossein Yousefinya, Camellia Torabizadeh, Farid Zand, Mahnaz Rakhshan, Mohammad Fararooei

**Affiliations:** 1 Instructor of Nursing, MSN, School of Nursing and Midwifery, Shiraz University of Medical Sciences, Shiraz, Iran. Email: yousefinya@sums.ac.ir University of Medical Sciences Shiraz University of Medical Sciences Shiraz Iran yousefinya@sums.ac.ir; 2 Associate Professor, Ph.D. of Nursing education, Department of Nursing, School of Nursing and Midwifery, Shiraz University of Medical Sciences, Shiraz, Iran. Email: torabik@sums.ac.ir. Corresponding Author. University of Medical Sciences Shiraz University of Medical Sciences Shiraz Iran torabik@sums.ac.ir; 3 Professor of Critical Care Medicine, MD, Department of Anesthesiology, School of Medicine, Shiraz University of Medical Sciences, Shiraz, Iran. Email: zandf@sums.ac.ir University of Medical Sciences Shiraz University of Medical Sciences Shiraz Iran zandf@sums.ac.ir; 4 Associate Professor, Ph.D. of Nursing education, Department of Nursing, School of Nursing and Midwifery, Shiraz University of Medical Sciences, Shiraz, Iran. Email: mzrakhshan@gmail.com University of Medical Sciences Shiraz University of Medical Sciences Shiraz Iran mzrakhshan@gmail.com; 5 Professor, Ph.D. of Epidemiology, Department of Epidemiology, School of Health, Shiraz University of Medical Sciences, Shiraz, Iran. Email: fararooei@yahoo.com University of Medical Sciences Shiraz University of Medical Sciences Shiraz Iran fararooei@yahoo.com

**Keywords:** clinical alarms, monitoring, intensive care units, observation, nurses., alarmas clínicas, monitoreo, unidades de cuidados intensivos, observación, enfermeras y enfermeros., alarmes clínicos, monitoramento, unidades de terapia intensiva, observação, enfermera e enfermeiros.

## Abstract

**Objective.:**

To evaluate the effects of application of a manual on the improvement of alarms management in Intensive Care Units (ICU).

**Methods.:**

This quasi-experimental study evaluated the effectiveness of the introduction into of a manual for alarm management and control in the ICU of a hospital in southeastern Iran. The intervention was a 4-hour workshop was on topics related to the adverse effects of alarms, standardization of ECG, oxygen saturation and blood pressure monitoring systems, and the use of ventilators and infusion pumps. Data were collected thorough 200 hours of observation of 60 ICU nurses (100 hours’ pre-intervention and 100 hours’ post-intervention). Response time, type of response, customization of alarm settings for each patient, the person responding to an alarm, and the cause of the alarm were analyzed. Alarms were classified into three types: false, true and technical.

**Results.:**

The results showed a statistically significant difference between the pre- and post-intervention frequency of alarm types, frequency of monitoring parameters, customized monitoring settings for patients, and individuals who responded to alarms. The percentage of effective interventions was significantly higher for all parameters after the intervention (46.9%) than before the intervention (38.9%).

**Conclusion.:**

The employment of a manual for management of alarms from electronic equipment in ICUs can increase the frequency of appropriate responses to alarms in these units.

## Resumen

**Objetivo.:**

Evaluar los efectos de la aplicación de un manual en el mejoramiento de la gestión de las alarmas en enfermeros las Unidades de Cuidados Intensivos -UCI-.

**Métodos.:**

Estudio cuasi- experimental en el que se evaluó la efectividad de la introducción de un manual en la práctica para la prevención y el control de alarmas en una UCI de un hospital del sudeste de Irán. Se realizó un taller de 4 horas de duración que incluía temas relacionados con los efectos adversos de las alarmas, la estandarización en los sistemas de monitoreo de ECG, saturación de oxígeno y presión arterial, y del uso de ventiladores y de bombas de infusión. Se realizaron 200 horas de observación a 60 enfermeras de la UCI (100 horas antes y 100 horas después de la intervención). Se analizó el tiempo de respuesta, el tipo de respuesta, la personalización de los ajustes de la alarma para cada paciente, la persona que responde a una alarma y la causa de la alarma. Las alarmas se clasificaron en tres tipos: falsas, verdaderas y técnicas.

**Resultados.:**

Los hallazgos mostraron diferencias estadísticamente significativas entre la frecuencia de los tipos de alarma antes y después de la intervención, la frecuencia de los parámetros de monitorización, los ajustes de monitorización personalizados para los pacientes y las personas que responden a las alarmas. El porcentaje de intervenciones efectivas para el total de parámetros fue significativamente mayor después de la intervención (46.9%) que antes de la misma (38.9%).

**Conclusión.:**

Los resultados del estudio muestran que el empleo del manual para la gestión de las alarmas de los equipos electrónicos en una UCI puede aumentar la frecuencia de respuestas adecuadas de los enfermeros de estos servicios.

## Resumo

**Objetivo.:**

Avaliar os efeitos da aplicação de um manual na melhoria do gerenciamento de alarmes em enfermeiros de Unidades de Terapia Intensiva -UTI-.

**Métodos.:**

Estudo quase-experimental no qual foi avaliada a eficácia da introdução na prática de um manual para prevenção e controle de alarmes em uma UTI de hospital no sudeste do Irã. Foi realizado um workshop de 4 horas que incluiu tópicos relacionados aos efeitos adversos dos alarmes, padronização dos sistemas de monitoração de ECG, saturação de oxigênio e pressão arterial e uso de ventiladores e bombas de infusão. Foram realizadas 200 horas de observação em 60 enfermeiras de UTI (100 horas antes e 100 horas após a intervenção). O tempo de resposta, o tipo de resposta, a personalização das configurações de alarme para cada paciente, a pessoa que responde a um alarme e a causa do alarme foram analisados. Os alarmes foram classificados em três tipos: falso, verdadeiro e técnico.

**Resultados.:**

Os resultados mostraram diferenças estatisticamente significativas entre a frequência dos tipos de alarme antes e depois da intervenção, a frequência dos parâmetros de monitoração, configurações de monitoração personalizadas para pacientes e pessoas que respondem aos alarmes. O percentual de intervenções eficazes para todos os parâmetros foi significativamente maior após a intervenção (46,9%) do que antes (38,9%).

**Conclusão.:**

Os resultados do estudo mostram que a utilização do manual para gerenciamento de alarmes de equipamentos eletrônicos em UTIs pode aumentar a frequência de respostas adequadas aos alarmes nos enfermeiros desses serviços.

## Introduction

Excluding the noises made by human interventions in clinical environments, almost 80% of the noise in intensive care units is due to electronic alarms. Because of their physiological impact, alarms lead to alarm fatigue.(1-3) Alarm fatigue is defined as incompetence at identifying or prioritizing alarms which, in turn, leads to inappropriate responses to alarms.(4) There are two reasons for alarm fatigue: becoming desensitized to alarms due to emotional overload and indifference to responses and confusion caused by alarms which results in distrust in accuracy of alarms.(5) Alarm fatigue not only increases emotional burden in healthcare personnel who are overexposed to alarms,(6,7) but also causes considerable stress to patients and healthcare providers.(8,9) Moreover, alarm fatigue can lead to delayed responses to alarms, readjustment of alarms to ranges which are unsafe for patients and eventually switching alarms silent or off.(9) As a growing problem with dire consequences, alarm fatigue was ranked by Emergency Care Research Institute (ECRI) as the top hazard among the 10 threats of technology in healthcare in 2012.(10) Alarm fatigue is a human error responsible for a large number of fatalities in hospitals. According to Food and Drug Administration, between 2002 and 2004, 237 deaths were caused by negligence of clinical alarms.(11,12) In a survey conducted by Federal Drug Administration over 4 months in 2010, 73 alarm-related deaths were investigated 33 of which were attributed to physiologic monitors.(13)

The purpose of clinical alarms is to enhance patients' safety and alert doctors and nurses to aberrations in patients' conditions. Also, when a patient's condition gets worse or a machine fails to function properly, alarms alert doctors and nurses.(12) It is a fact that nurses are the best monitors of patients and physiologic monitors are simply for further assurance of the doctors and nurses who use them; however, the issue of false positive alarms remains a serious unsolved problem with many grave consequences.(14) False alarms are cry wolves which lead nurses to ignore alarms or respond to repetitive alarms with delay. Furthermore, alarms can prevent nurses from planning effectively and performing their tasks properly and distract them in other ways.(14) Studies show that the majority of alarms are false.(3,14) The results of 371 hours of monitoring in a study showed that 1762 alarms (99.4%) were false alarms.(15) In their study, Graham and Cvach have recorded 942 alarms per day or 1 critical alarm every 92 seconds, while other researchers report 6 to 16 alarms per hour.(16) Delay settings on SpO_2_ (peripheral capillary oxygen saturation) alarm systems up to 15 seconds or 19 seconds,(17) can reduce the frequency of alarms to 50% or 70% respectively; thus, in the case of lack of oxygen saturation in short periods, the number of alarms is decreased.(10) Setting alarm ranges according to each patient's condition can also reduce the frequency of alarms, thus decreasing alarm fatigue.(17)

Considering the results of studies regarding the consequences of alarm fatigue and given the fact that, so far, few studies have addressed alarm fatigue management, the present study is an attempt at exploring the effects of development and introduction of a manual for alarm management for intensive care unit nurses.

## Methods

This study was approved by the Research Ethics Committee of Shiraz University of Medical Sciences located in Southwest of Iran (IR.SUMS.REC.1393.S7362), data were collected in 2017. The present study is a quasi-experimental work of research conducted in two stages: development of a manual for management of alarms from clinical equipment and introduction of the manual. Since a nurse's response to an alarm is an occurrence which may happen at any time in a hospital unit and is not considered a unique event, i.e. a nurse's response to an alarm is independent of the changes specific to a period, in the present study, considering the nature of the behavior under study, the researchers employed the methods of observation and time sampling.(18) The sample consisted of 200 hours of observation in an intensive care unit-100 hours before the intervention and 100 hours after the intervention. Accordingly, the nurses' responses to alarms were observed during different days and shifts (morning, evening and night) for 6.5 hours per shift.

To perform the observation, two of the researchers who were completely familiar with intensive care units would randomly select a shift every day and record the nurses' responses to alarms. For one month, data were collected from morning, afternoon, and evening shifts by the two researchers using a checklist. The study population consisted of all the practicing nurses in the general intensive care unit of the largest hospital in the south of Iran. Sampling was based on the census method and the subjects who met the inclusion criteria of the study and filled out the informed consent form were included. The inclusion criteria were to be a permanent nursing staff member in the intensive care unit, to not have participated in a similar work of research, and to be willing to participate. The exclusion criterion was failing to attend one of the education sessions (none of the nurses were excluded).

Data Collection. The researchers developed the monitoring system alarms data collection form based on a literature review and the results of previous studies. The data collection form includes items about type of monitoring parameter, type of alarm, response time to alarm (the time lapse between the moment an alarm starts and the moment a response is made to it), type of response, personalization of alarm settings for each patient, the individual who responds to an alarm, and cause of alarm. Alarms were classified into three types: false, true, and technical. A false alarm is one which is detected and recorded by the monitoring system as a physiologic occurrence, while actually nothing has occurred. A true alarm is a correct detection of a deviation in parameter settings recorded by the monitoring system. A technical alarm is defined as an alarm which informs nurses of a problem in the functioning of a monitoring system, e.g. disconnection of a machine from the power supply and disconnection of the pipes and other gears attached to patients. The equipment dealt with in the present study were the entire vital signs monitoring systems, cardiac monitoring systems, ventilators and syringe pumps. To increase the validity of the findings, the data collected from the first 30 hours of observations were excluded and the data obtained during the following 200 hours of observations were analyzed. To test the reliability of a data collection form (in order to reduce the possibility of errors and increase consistency in the collection of data), the observers are required to be in the same environment and record the same event simultaneously. Accordingly, the two researchers who were responsible for data collection in the present study were placed in a simulated environment where they were shown the same video of patient monitoring systems and answered the questions on the form about the alarms. Subsequently, SPSS v. 21 was used to measure the consistency between the data collected on the two forms. The consistency was found to be 0.88.

The manual for alarm management in intensive care units was developed as a protocol by three intensive care specialists, an intensive care unit head nurse and two experienced staff members, and the research team during three two-hour focus group sessions. The manual includes sections on introduction to alarms, the consequences of alarms, and methods of controlling and preventing alarms. The manual was introduced to the ICU personnel over a six-session workshop (Table 1). Lasting for 4 hours, each session was directed by experienced professors in the field of intensive care. After the introduction of the manual for alarm management, the ICU personnel were asked to adjust themselves to the instructions they had received for two weeks. In the first week of the initiation, the personnel's inquiries were dealt with; in the second week, the personnel were allowed to use the manual on their own. Next, as of the fifteenth day, data were collected again in the intensive care unit using the monitoring system alarms data collection form.

**Table 1:** Content of the workshops

Session	Goal	Content
1	Introduction to alarms and their adverse effects	Discussing the role of alarms in the safety of patients
1	Introduction to alarms and their adverse effects	Discussing the methods of controlling and preventing alarms
2	Standardization of ECG monitoring systems	A review of the content of the previous session
2	Standardization of ECG monitoring systems	Empowerment of the personnel by educating them in how to properly prepare the skin for ECG electrodes, daily replacement of electrodes, and modification of alarm parameters and thresholds of ECG monitors, among others
3	Standardization of SpO_2_ and blood pressure monitoring systems	A review of the content of the previous session
3	Standardization of SpO_2_ and blood pressure monitoring systems	Modification of delay and threshold settings in SpO_2_ monitoring systems
3	Standardization of SpO_2_ and blood pressure monitoring systems	Modification of threshold settings in blood pressure monitoring systems
4	Empowerment and education of personnel in using ventilators	A review of the content of the previous session
4	Empowerment and education of personnel in using ventilators	Modification of settings of a ventilator, including respiratory rate, tidal volume, positive end-expiratory pressure, and respiratory tract pressure
5	Standardization of syringe pumps and infusion pumps	A review of the content of the previous session
5	Standardization of syringe pumps and infusion pumps	Modification of threshold settings in syringe pumps and infusion pumps
5	Standardization of syringe pumps and infusion pumps	Introduction to alarm types of syringe pumps and infusion pumps
6	Starting an inter-professional team	A review of the content of the previous session
6	Starting an inter-professional team	Creating an inter-professional team in the hospital which oversees alarm-related issues, including policy development and introduction of new methods

Abbreviations: SPO_2,_ Peripheral capillary oxygen saturation; ECG, Electrocardiography

Data Analysis. The collected data were analyzed using SPSS v. 21. The central indexes and the distribution of the data were measured using descriptive statistics, including mean, standard deviation, frequency, and percentage based on the type of variable (quantitative or qualitative). In order to assess the efficacy of the interventions, the researchers employed the statistical tests of paired sample t-test, independent t-test, and Chi-square. The assumption that the variables were normal was tested using Kolmogorov-Smirnov test; if the assumption was disproved, nonparametric tests, including Mann-Whitney test, Wilcoxon test, Fisher's exact test, McNemar test, and sign test would be used. The significance level for the tests was set at 0.05 and the confidence interval for the calculation of point estimations was 95%.

## Results

In the present study, 60 nurses with the average age of 34.36±6.42 years and work experience of 9.69±7.44 years participated. 63.3% of the participants were female and 36.7% were male. 83.4% had a bachelor's degree and 16.6% had a master's degree. Also, 26.6% of the participants were permanent employees, 36.6% were fixed-term employees, 16.6% were contractual employees, and 20% were trainees. The results showed a significant reduction (53.8%) in the frequency of false alarms following the intervention. Also, the results of the Chi-square test showed a statistically significant difference between the pre- and post-intervention alarm types (*p*<0.001). As only one technical alarm was observed and because such alarms are in fact true alarms, the number of technical alarms was considered together with the true alarms and the tests were conducted between the two groups of false and true alarms (Table 2).

**Table 2:** Frequency distribution of the types of alarms before and after the intervention

Type of alarm	Before	After	Percentage change
Type of alarm	*n* (%)	*n* (%)	Percentage change
False	552 (79.5)	255 (48.9)	-53.8
True^*^	141 (20.4)	262 (50.1)	85.81
Technical	1 (0.1)	5 (1)	4
Total	694	522	*p*<0.001

* Technical and true alarms were merged due to the small number of technical alarms, these alarms were combined with the true alarms in statistical calculations.

The results of Fisher's exact test showed a statistically significant difference between the pre- and post-intervention frequency distribution of monitoring parameters (*p*<0.001). The highest and lowest impacts of the introduction of the manual belonged to RR (Respiratory Rate) monitoring (with a 64.7% reduction in frequency of alarms) and NIBP monitoring (with a 19.8% reduction in frequency of alarms) respectively (overall *p* <0.05) (Table 3).

**Table 3:** Frequency distribution of monitoring parameters before and after the intervention

Type of parameter	Type of alarm	Before	After	***p*-value**
		***n* (%)**	***n* (%)**	
SPO_2_	False	228 (32.85)	110 (21.7)	<0.001
SPO_2_	True	37 (5.33)	89 (17.4)	<0.001
NIBP	False	51(7.34)	29 (5.55)	0.037
NIBP	True	40 (5.76)	44 (8.42)	0.037
ECG	False	140 (20.17)	66 (12.64)	<0.001
ECG	True	26 (3.74)	58 (11.11)	<0.001
Ventilator	False	14 (2.01)	40 (7.66)	0.005
Ventilator	True	33 (4.75)	31 (5.93)	0.005
RR	False	107 (15.41)	6 (1.14)	<0.001
RR	True	5 (0.72)	27 (5.17)	<0.001
S.P	False	12 (1.72)	4 (0.76)	0.01
S.P	True	1 (0.14)	5 (0.95)	0.01
Total	False	552 (79.5)	255 (48.9)	<0.001
Total	True	142 (20.5)	267 (51.1)	<0.001

Abbreviations: SPO_2,_ Peripheral capillary oxygen saturation; NIBP, Non Invasive Blood Pressure; ECG, Electrocardiography; RR, Respiratory Rate; S.P, Syringe pump

The results of Fisher's exact test showed that the difference between the pre- and post-intervention types of response to alarms was not statistically significant. The most and least effective responses to alarms after the intervention belonged to SpO_2_ (16.47%) and syringe pumps (0.5) was significant for the total of parameters. However, the percentage of effective interventions was significantly higher for all parameters after the intervention (46.9%) than before the intervention (38.9%) with a *p*-value of 0.005 (Table 4).

**Table 4:** A comparison between pre- and post-intervention types of response to alarms

Type of parameter	Type of response	Before	After	***p*-value**
		***n* (%)**	***n* (%)**	
SPO_2_	Effective	95 (13.68)	86 (16.47)	0.17
SPO_2_	Ineffective	170 (24.49)	116 (22.22)	0.17
NIBP	Effective	53 (7.63)	50 (9.58)	0.24
NIBP	Ineffective	40 (5.76)	26 (26)	0.24
ECG	Effective	75 (10.8)	68 (13.5)	0.12
ECG	Ineffective	91 (13.11)	57 (10.92)	0.12
Ventilator	Effective	33 (4.75)	30 (5.75)	0.73
Ventilator	Ineffective	13 (1.87)	10 (1.91)	0.73
RR	Effective	9 (1.29)	8 (1.55)	0.44
RR	Ineffective	102 (14.69)	61 (11.7)	0.44
S.P	Effective	5 (0.72)	3 (0.5)	0.71
S.P	Ineffective	8 (1.15)	7 (1.35)	0.71
Total	Effective	270 (38.9)	245 (46.93)	0.005
Total	Ineffective	424 (61.1)	277 (53.07)	0.005

Abbreviations: SPO_2,_ Peripheral capillary oxygen saturation; NIBP, Non Invasive Blood Pressure; ECG, Electrocardiography; RR, Respiratory Rate; S.P, Syringe pump

Even though the results of Wilcoxon test indicated a statistically significant difference (*p*-value=0.001) between the pre- and post-intervention personalized monitoring system settings for each patient, the majority of the alarms and parameters (85.8%) remained without personalized settings. The greatest and smallest numbers of personalized settings for monitoring each patient as implemented by the nurses belonged to SpO_2_ monitoring and ventilators respectively (Table 5).

**Table 5:** A comparison between pre- and post-intervention personalized monitoring settings for each patient

Type of parameter	Before	After	***p*-value**
	***n* (%)**	***n* (%)**	
SPO_2_	0 (0)	26 (5)	<0.001
NIBP	0 (0)	23 (4.4)	<0.001
HR	0 (0)	13 (2.5)	<0.001
Ventilator	0 (0)	12 (2.3)	0.001
None	694 (100)	448 (85.8)	<0.001
Total	694	522	

Abbreviations: SPO_2,_ Peripheral capillary oxygen saturation; NIBP, Non Invasive Blood Pressure; HR, Heart Rate; S.P, Syringe pump

Based on the results of Fisher's exact test, there was a statistically difference between the pre- and post-intervention types of individual who responded to alarms. Before the intervention, the nurses responded to 43.1% of the alarms, while after the intervention, they responded to 62.1% of the alarms. In other words, in the post-intervention stage, the nurses were more responsive to the alarms from the patients' monitoring systems. Besides the nurses, the personnel consisted of the doctors, medical equipment technicians, and the cleaning staff. In the present study, the doctors were never seen to respond to an alarm in any way (Table 6).

**Table 6:** Frequency of pre- and post-intervention types of individual who responded to alarms

Type of parameter	Type of alarm	Before	After	***p*-value**
		***n* (%)**	***n* (%)**	
SPO_2_	Nurse	110 (15.8)	130 (24.9)	<0.001
SPO_2_	Nursing assistant	31 (4.4)	11 (2.1)	<0.001
SPO_2_	Others^*^	21 (3)	3 (0.5)	<0.001
SPO_2_	No response	103 (14.9)	58 (11.1)	<0.001
NIBP	Nurse	61 (7.8)	63 (12)	0.01
NIBP	Nursing assistant	8 (1.2)	1 (0.2)	0.01
NIBP	Others	4 (0.6)	0 (0)	0.01
NIBP	No response	18 (2.6)	12 (2.3)	0.01
ECG	Nurse	74 (10.7)	78 (14.9)	0.008
ECG	Nursing assistant	24 (3.5)	6 (1.1)	0.008
ECG	Others	1 (0.1)	0 (0)	0.008
ECG	No response	67 (9.7)	41 (7.9)	0.008
Ventilator	Nurse	34 (4.9)	34 (6.5)	0.15
Ventilator	Nursing assistant	3 (0.4)	1 (0.2)	0.15
Ventilator	Others	2 (0.3)	0 (0)	0.15
Ventilator	No response	8 (1.2)	5 (1)	0.15
RR	Nurse	10 (1.4)	11 (2.1)	0.15
RR	Nursing assistant	2 (0.3)	0 (0)	0.15
RR	Others	0 (0)	0 (0)	0.15
RR	No response	100 (14.4)	58 (11.1)	0.15
S.P	Nurse	10 (1.4)	8 (1.5)	0.86
S.P	Nursing assistant	0 (0)	0 (0)	0.86
S.P	Others	1 (0.1)	1 (0.2)	0.86
S.P	No response	2 (0.3)	1 (0.2)	0.86

* The groups of others, nursing assistant, and no response were merged. Abbreviations: SPO_2,_ Peripheral capillary oxygen saturation; NIBP, Non Invasive Blood Pressure; ECG, Electrocardiography; RR, Respiratory Rate; S.P, Syringe pump

## Discussion

In this study, 1216 alarms were recorded over 200 hours of observation, with the mean of 6.08 alarms per hour. Of the 1216 recorded alarms, 66.3% were false alarms, 33.1% were true alarms, and 0.4% were technical alarms. In their study, Graham et al. recorded 1 clinically significant alarm every 92 seconds.(16) Baillargeon reports that of the 174 alarms recorded in her study, 44.8% were false alarms, 47.7% were true alarms, and 7.4% were technical alarms.(6) In the study of Inokuchi *et al*. only 6.4% of the 11591 recorded alarms were found to be clinically significant; 93.6% were false alarms and 0.02% were technical alarms.(19) The findings of the present study are consistent with those of the studies of Graham et al., and Inokuchi *et al*. but do not agree with the results of Baillargeon's study, which may be due to the shorter time span of Baillargeon's study or the fact that her study was conducted in an internal unit where telemetry monitoring was used.

Before the introduction of the educational intervention, 694 alarms were recorded, 79.5% of which were false alarms, 20.3% were true alarms, and 0.1% were technical alarms. After the intervention, of the 522 recorded alarms, 48.9% were false alarms, 50.2% were true alarms, and 1% were technical alarms. The 53.8% reduction in the occurrence of false alarms after the intervention can be attributed to the education which the nurses received in the workshops. Graham et al. report a 43% posttest reduction in the frequency of physiologic alarms.(16) In their study of the effect of daily replacement of ECG electrodes on the alarms of electrocardiogram monitors in CCU (Coronary Care Unit) and MPCU (Medical Progressive Care Unit), Cvach *et al.* report that the total number of daily alarms per bed decreased by 47% in MPCU and by 44% in CCU. Moreover, the number of technical alarms in MPCU and CCU decreased by 34% and 45% respectively; the number of audio alarms in MPCU and CCU decreased by 52% and 46% respectively; finally, the number of alert alarms in MPCU and CCU decreased by 47% and 46% respectively. However, the frequency of high-priority (critical) alarms did not change.(13) The results of the studies of Graham et al., and Cvach *et al.* are consistent-the greater reduction in the post-intervention number of false alarms in the study of Cvach *et al*. can be due to the fact that they included the entire monitoring systems in their study.

The results of this study showed that the difference between the pre-intervention response time (51.9±67.21 seconds) and the post-intervention response time (30.8±15.09 seconds) is statistically significant. In Baillargeon's study, alarm response time ranges between 1 minute and 20 seconds and 10 minutes, with the mean being 7.01 minutes(6) which is far more than the response time in the present study. In the present study, before the intervention, the longest response time was for SPO_2_ monitoring (17.3 minutes). After the intervention, the longest response time, at 1.6 minutes, was for ECG (Electrocardiography), RR, and SPO_2_ monitoring.

Of the 1216 alarms recorded in the present study, 38.4% were for SPO_2_, 23.9% were for ECG, 14.8% were for RR, 13.7% were for NIBP, 7.2 % were for ventilator, and 1.9% were for syringe pump monitoring systems. The difference between the pre- and post-intervention frequency distributions of monitoring parameters was not statistically significant (*p*-value=0.92). In other words, even though the number of false alarms (as the primary source of alarm fatigue in nurses) decreased as a result of the educational interventions, the change in the frequency of monitoring parameters after the intervention was not statistically significant, which can be attributed to the higher frequency of true alarms from monitoring parameters in the post-intervention stage. However, in their study conducted at the Johns Hopkins Hospital, Taenzer *et al.* report a 63% reduction in the number of alarms after lowering the threshold of SPO_2_ monitor.(20) In the study of Cvach *et al*. the frequency of alarms related to arrhythmia and RR electrodes dropped in CCU and MPCU by 74% and 60% respectively for arrhythmia and by 65% and 36% for RR.(13)

According to the results of the present study, 59.53% of the alarms occurred during the morning shifts. The majority of the alarms were recorded during the morning shifts. The increased workload of nurses in the morning, the execution of many of the clinical procedures in the morning, doctors' rounds, and the presence of patients' visitors during morning visiting hours can account for the higher frequency of alarms in morning shifts. The findings of our study show a statistically significant difference between the nurses' pre- and post-intervention types of response to alarms: 42.35% of the alarms were responded to effectively and 57.65% received ineffective responses. Before the intervention, 38.9% of the alarms were responded to effectively and 61.1% were responded to ineffectively, while after the intervention, 46.93% of the alarms were responded to effectively and 53.06% were responded to ineffectively. Effective responses were those in which the type of alarm was checked, the signs were interpreted by a nurse and, subsequently, steps were taken to deal with the cause of the alarm or the doctors were informed. Ineffective responses were characterized by silencing alarms, delay in responding to alarms, ignoring alarms or deactivating them without investigating the cause of the alarm. According to Harris et al. the number of false and nuisance alarms is a determining factor in clinical alarm response time.(21) Jennings reports that when the frequency of nuisance alarms is high, the medical staff is more likely to ignore relevant alarms.(22) Similarly, Graham et al. state that positive false alarms or nuisance alarms can result in delayed response time or reduce the likelihood of nurses' responding to alarms.(16)

In this study, before the intervention, none of the alarms were personalized by the nurses for the monitoring of patients and all the alarms were on default settings. However, after the intervention and the visual and practical education on the use of monitors in the workshops, the situation improved: of the 522 alarms recorded in the post-intervention stage, 14.17% of them made the nurses examine the sensitivity range of the monitors and alter them if necessary. Even though the difference between the pre- and post-intervention personalization of settings for the monitoring of each patient was statistically significant (P-value=0.001), a large number (85.8%) of alarms and parameters remained unchanged. These findings are consistent with the results of the study of Graham et al.: they found that modification of the default settings of monitors and informing nurses about the significance of personalization of monitor parameters can help reduce excess alarms.(16)

Based on the findings of this study, 51.23% of the alarms were responded to by nurses, 7.15% were responded to by nursing assistants, and 2.71% were responded to by others, including patients' visitors and the cleaning staff. 38.89% of the alarms were never responded to. It was also noticed that the cleaning staff occasionally silenced alarms that went off while they were cleaning the rooms and equipment. Before the intervention, 43.1% of the alarms were responded to by the nurses, while after the educational intervention, the nurses responded to 62.1% of the alarms. The results of the statistical tests show a significant difference (*p*-value=0.007) between the pre- and post-intervention frequency of responders to alarms: the nurses became more responsive. Even though the responsiveness of the nurses in charge of checking the clinical conditions of patients and the monitoring parameters improved in the present study after the intervention, the number of alarms which did not receive a response was still high (33.5%).

Limitations. One of the limitations of the study was its use of the observation method for data collection: changes in the observers' behavior, perception errors, and environmental factors can adversely affect the reliability of the data. To reduce the impact of such factors, the researchers excluded the results of the first 5 observations. Also, to minimize the impact of the observers' presence, the researchers wore the same uniform as the personnel, established a friendly relationship with the personnel, and entered and exited the intensive care unit at the same time as the personnel.

## Conclusion

According to the results of the field observations, aside from nurses who are considered the primary responders to alarms, other individuals, including the cleaning staff and even patients' visitors, occasionally respond to alarms. Application of the manual for management of alarms in intensive care units can prove useful in decreasing the number of alarms and, consequently, their negative consequences. Considering the broadness of the issue of alarm fatigue and its serious consequences for both patients and nurses, it is suggested that alarm fatigue be measured in nurses and the necessary steps, including educational workshops and classes, be taken to deal with this issue.
